# Klonale Hämatopoese – Bedeutung für die Zelltherapie

**DOI:** 10.1007/s00108-022-01403-y

**Published:** 2022-09-23

**Authors:** Raphael Teipel, Malte von Bonin, Friedrich Stölzel, Johannes Schetelig, Christian Thiede, Martin Bornhäuser

**Affiliations:** 1 Medizinische Klinik und Poliklinik 1, Universitätsklinikum Carl Gustav Carus an der TU Dresden, Fetscherstr. 74, 01307 Dresden, Deutschland; 2 DKMS Clinical Trials Unit, Dresden, Deutschland; 3 AgenDix, Gesellschaft für angewandte molekulare Diagnostik mbH, Dresden, Deutschland; 4 Nationales Centrum für Tumorerkrankungen Dresden (NCT/UCC), Dresden, Deutschland

**Keywords:** Autologe Stammzelltransplantation, Allogene Stammzelltransplantation, CAR-T-Zell-Therapie, Sekundärmalignome, Inflammation, Stem cell transplantation, autologous, Stem cell transplantation, allogeneic, CAR T‑cell therapy, Neoplasms/secondary, Inflammation

## Abstract

Der Nachweis klonaler Hämatopoese (CH) bei Patient*innen mit hämatologischen Neoplasien, die mit einer zellulären Therapie behandelt werden, ist häufig. Zu den gängigen, in der klinischen Routine verwendeten zellulären Therapieverfahren zählen die autologe und allogene Stammzelltransplantation (SZT) und seit Kurzem die CAR-T-Zell-Therapie (*CAR* chimärer Antigenrezeptor). Alle drei Verfahren unterscheiden sich fundamental im Hinblick auf Gewinnung, Verarbeitung und Einsatz des jeweiligen Zellprodukts. Deshalb ist die Bedeutung der CH in Bezug auf das jeweilige Therapieverfahren grundsätzlich unterschiedlich zu bewerten und einzuordnen. Bei der autologen SZT trägt das Ausmaß der zytotoxischen Vortherapie maßgeblich zur hohen CH-Prävalenz bei. Der klinisch bedeutsamste Aspekt ist hier die Entwicklung von Sekundärneoplasien aus einer präexistenten CH sowie das potenziell erhöhte Risiko kardiovaskulärer Nebenwirkungen. Bei der allogenen SZT bestimmt die Auswahl der Spender*innen im Hinblick auf das Alter die Wahrscheinlichkeit für das Vorliegen einer CH. Die Entwicklung von Sekundärmalignomen spielt verglichen mit der autologen SZT nur eine untergeordnete Rolle. Vielmehr scheinen die Induktion eines Graft-versus-Host(GvH)- bzw. eines Graft-versus-Leukemia(GvL)-Effekts und deren Einfluss auf Rezidivfreiheit und Überleben von möglicher klinischer Relevanz. Die CAR-T-Zell-Therapie ist in ihrer Wirkungsweise und in Bezug auf das Nebenwirkungsprofil eng verknüpft mit Inflammationsreaktionen. Auch hier besteht ein potenzieller Zusammenhang zwischen CH sowie Wirkung und Nebenwirkung einer CAR-T-Zell-Therapie. Erste Daten berichten über eine hohe Prävalenz von CH bei Patient*innen vor CAR-T-Zell-Therapie und deuten auf eine erhöhte Rate an inflammatorischen Nebenwirkungen hin, wenngleich sich bisher kein negativer Effekt auf das Überleben zeigt.

## Abstract (en)

The detection of clonal hematopoiesis (CH) in patients with hematologic neoplasms who are undergoing a cellular therapy is common. The most frequently used cellular therapy procedures include autologous and allogeneic hematopoietic stem cell transplantation (HSCT) and, more recently, chimeric antigen receptor (CAR) T‑cell therapy. All three procedures differ fundamentally in terms of harvesting and manufacturing aspects as well as usage of the respective cell product. Therefore, the importance of CH in relation to the respective treatment method must be evaluated and assessed differently. In autologous HSCT, the extent of previous cytotoxic therapy significantly contributes to the high prevalence of CH. The clinically most important aspect is the development of secondary neoplasms from a pre-existing CH clone and the potential risk for enhanced cardiovascular side effects. In allogeneic HSCT, the donor selection with respect to the age largely determines the probability for the presence of CH. In this setting, the development of secondary malignancies only plays a minor role compared to the autologous HSCT. In fact, the induction of a graft versus host (GvH) or a graft versus leukemia (GvL) effect and its influence on progression-free and overall survival seem to be of possible clinical relevance. The CAR T‑cell therapy is closely linked to inflammatory reactions regarding its mode of action and the associated side effects. In this context CH might be closely linked to the effectiveness and side effects of the CAR T‑cell therapy. Initial data reported a high prevalence of CH in patients before CAR T‑cell therapy and indicated an increased rate of inflammatory side effects, although no negative effect on survival has yet been demonstrated.

Die verschiedenen Verfahren der Zelltherapie bei Patient*innen mit hämatologischen Neoplasien unterscheiden sich wesentlich in Bezug auf ihre Wirkungsweise, Ursprungsquelle und Herstellung des Zellprodukts sowie ihr Nebenwirkungsprofil. Dies hat auch spezielle Implikationen für den Nachweis und Ursprung einer klonalen Hämatopoese (CH) im jeweiligen Zellprodukt sowie die nachfolgende Transplantation/Reinfusion und Expansion der (klonalen) hämatopoetischen Zellpopulationen in den entsprechenden Empfänger*innen. Im Folgenden werden die aktuell im klinischen Alltag am häufigsten eingesetzten Verfahren diskutiert:

- Autologe Stammzelltransplantation (SZT)
- Allogene SZT
- CAR-T-Zell-Therapie (*CAR* chimärer Antigenrezeptor)

Dabei wird auf die Bedeutung der CH und deren Auswirkungen für das jeweilige Verfahren eingegangen.

## Klonale Hämatopoese im Kontext der autologen Stammzelltransplantation

**Figure 1:**
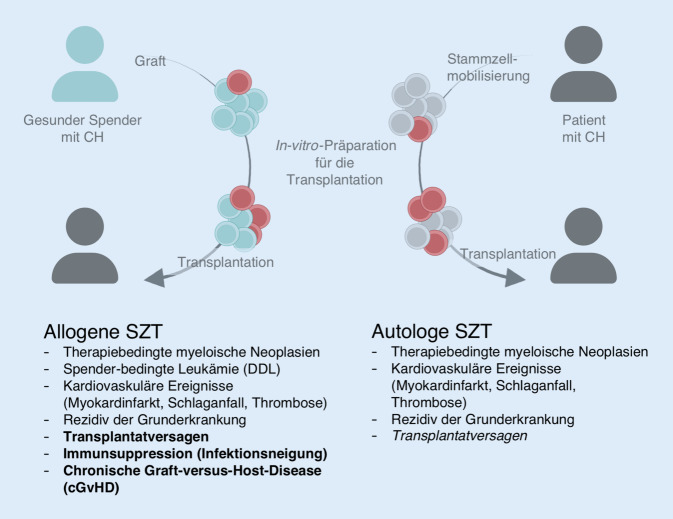

Das Verfahren der autologen SZT nach vorangegangener Hochdosischemotherapie stellt in der Behandlung bestimmter hämatoonkologischer Neoplasien, allen voran maligner Lymphome und des multiplen Myeloms, ein gängiges Therapieprinzip dar. Hierbei wird die Transplantation entweder als Teil der Primärtherapie (multiples Myelom) oder im Rezidiv (beispielsweise maligne Lymphome, Keimzelltumoren) angewendet (Abb. 1).

Bei Patient*innen mit hämatologischen Neoplasien findet sich häufig eine erhöhte Prävalenz von CH

Bei Patient*innen mit hämatologischen Neoplasien findet sich häufig eine erhöhte Prävalenz von CH verglichen mit der altersentsprechenden Kohorte gesunder Menschen [3, 10, 13, 28]. In Proben von stark vorbehandelten Patient*innen mit Lymphomen und multiplem Myelom konnten bei bis zu 30 % der Patient*innen vor autologer hämatopoetischer SZT entsprechende Mutationen gefunden werden. Als entscheidender Einflussfaktor kann hierbei die stattgehabte Exposition gegenüber zytotoxischen Substanzen, allen voran klassischen Chemotherapeutika angesehen werden. Dies kann zum einen das Auftreten von De-novo-Mutationen fördern und zum anderen die Selektion und Expansion bestimmter Klone mit solchen Mutationen begünstigen. Je nach Sensitivität der Methodik und erreichbarer Detektionstiefe kann auch schon bei weniger stark vorbehandelten Patient*innen eine erhöhte Mutationslast gefunden werden [17]. Vor allem das Vorliegen bestimmter Treibermutationen und die Größe des jeweiligen Klons scheinen für den Einfluss der CH in diesem Kontext von erheblicher Bedeutung zu sein [27].

### Stammzellgewinnung

Die erfolgreiche Gewinnung der autologen Stammzellpräparate setzt die ausreichende Mobilisierung hämatopoetischer Stammzellen aus der Knochenmarknische ins periphere Blut voraus. Dies wird hauptsächlich durch die Gabe von Granulozyten-koloniestimulierendem Faktor (G-CSF), entweder allein (sogenannte Steady-State-Mobilisierung) oder in Kombination mit Chemotherapeutika, erreicht. Bei schlechter Mobilisierung wird zusätzlich Plerixafor, ein Chemokinrezeptorantagonist, eingesetzt, um die Ausbeute hämatopoetischer Stammzellen zu erhöhen. Bisher existieren keine systematischen Daten, inwieweit die verschiedenen Mobilisierungsstrategien die Transplantatzusammensetzung hinsichtlich des Anteils von CH beeinflussen. Es besteht beispielsweise die Möglichkeit, dass eine Stammzellapherese nach Einsatz einer Mobilisierungschemotherapie hämatopoetische Stammzellen selektioniert, die Mutationen in DNA-Reparatur-Mechanismen aufweisen.

Relevanter ist allerdings die Frage, ob das Vorliegen einer CH direkten Einfluss auf den Mobilisierungsprozess besitzt und somit das Sammelergebnis quantitativ beeinflusst. Mehrere Studien vermuten einen negativen Zusammenhang mit der Quantität des Stammzellgrafts [1, 11, 24], wobei die Datenlage hier nicht eindeutig ist [3, 19].

### Stammzelltransplantation

Die klinisch entscheidende Fragestellung ist und bleibt, inwieweit das Vorliegen einer CH die Ergebnisse der autologen SZT beeinflussen kann. Das Vorhandensein einer CH im autologen Transplantat wurde in einigen retrospektiven Studien mit einem reduzierten Gesamtüberleben [10, 17] in Verbindung gebracht. Vor allem bei Patient*innen mit malignen Lymphomen und einer ausgedehnteren stattgehabten Zytostatikaexposition zum Zeitpunkt der autologen SZT zeigte sich eine überproportionale Häufung von Mutationen im Bereich von DNA-Reparatur-Genen (*TP53, PPM1D*). Bei diesen Patient*innen war die Rate an therapieassoziierten myeloischen Neoplasien signifikant höher als bei Vergleichspatienten ohne Nachweis einer CH [13, 24]. Gibson et al. berichteten eine kumulative 10-Jahres-Inzidenz von 14,3 %. Allerdings tragen Sekundärneoplasien nur teilweise zum beschriebenen schlechteren Gesamtüberleben dieser Patient*innen bei. Daneben scheinen andere Komorbiditäten (unter anderem kardiovaskuläre Ereignisse, Infektionen, verzögertes Engraftment), die ebenfalls im Rahmen von CH verstärkt berichtet werden, eine relevante Rolle zu spielen [10, 13, 19, 24].

Im Gegensatz dazu konnte bei Patient*innen mit multiplem Myelom und Nachweis einer CH kein erhöhtes Risiko der Entwicklung einer therapieassoziierten Sekundärneoplasie gesehen werden, bei allerdings divergentem Mutationsspektrum. Hier spielt möglicherweise der Zeitpunkt der Mutationsanalyse im Hinblick auf Art und Dauer der vorangegangenen Therapie eine Rolle. Dennoch war das Vorliegen von CH in dieser Studie mit einem schlechteren Überleben assoziiert, vorwiegend durch ein erhöhtes Rezidivrisiko. Interessanterweise konnte dieser negative Einfluss von CH auf das Überleben durch eine Lenalidomiderhaltungstherapie nach autologer SZT aufgehoben werden [17].

### Resümee und Ausblick

Die Prävalenz von CH zum Zeitpunkt der autologen Transplantation hängt hauptsächlich vom Alter der Patient*innen und der Intensität der vorherigen Behandlung ab. Die autologe SZT stellt wie andere Verfahren, die Stress auf die Hämatopoese ausüben, einen Risikofaktor für die Selektion von CH dar. Der Beitrag verschiedener Teilaspekte, beispielsweise der Mobilisierung, Konditionierung und Transplantation, bleibt jedoch vage.

Der Nachweis von CH sollte die Therapieentscheidung zur autologen SZT derzeit nicht beeinflussen

Die Auswirkungen von CH auf das Outcome können zwischen verschiedenen Kohorten variieren, unter anderem abhängig von der Art und Intensität der Behandlung, den betroffenen Genen und der Mutationslast. Bis heute scheint die Wirksamkeit der autologen SZT durch das Vorhandensein von CH unbeeinflusst zu sein. Daher sollte der Nachweis von CH derzeit keinen Einfluss auf die klinische Entscheidungsfindung im Zusammenhang mit der autologen SZT haben.

## Klonale Hämatopoese im Kontext der allogenen Stammzelltransplantation

Bei der allogenen SZT erfolgt eine Übertragung hämatopoetischer Stammzellen von fremden Spender*innen (Familien- oder Fremdspender*innen) mit dem Ziel, eine immunologische Wirksamkeit gegen die vorliegende Neoplasie der Empfänge*innen zu induzieren (Abb. 1). Eine seltene, aber relevante Komplikation im Rahmen einer allogenen SZT ist die Entwicklung einer sekundären myeloischen Neoplasie (myelodysplastisches Syndrom oder akute myeloische Leukämie, zusammengefasst als „Spenderleukämie“), die sich aus originären hämatopoetischen Zellen der Spender im Mikromilieu der Empfänger*innen entwickelt. Es ist denkbar, dass die Spenderleukämie in diesen Fällen aus einer schon vorbestehenden CH der Spender*innen entsteht. In einer größeren europäischen Auswertung lag die Häufigkeit einer Spenderleukämie bei 0,8 %. Retrospektiv konnte hier in 28 % aller Fälle im Transplantat der Nachweis einer CH erbracht und somit die Entstehung der Mutation bereits in der Spender*in (*vor* der Stammzellspende) nachgewiesen werden [4].

### Einfluss der Spenderauswahl

Im Gegensatz zur autologen SZT, bei der die Transplantate zum Großteil aus Patient*innen > 50 Jahre gewonnen werden, werden allogene SZT aus nichtverwandten Spender*innen in der Regel mit Zellprodukten jüngerer, gesunder Spender*innen durchgeführt. Hier besteht eine altersentsprechend sehr niedrige Prävalenz (< 1 %) einer klonalen Hämatopoese von unbekanntem Potenzial (CHIP; [7, 14, 29]).

Eine longitudinale Analyse von 22 älteren Patient*innen (Median 67 Jahre zum Zeitpunkt der Transplantation), die eine allogene SZT von jüngeren Stammzellspender*innen (Median 34 Jahre) erhalten hatten, ergab ein sehr niedriges Risiko der Entwicklung einer De-novo-CH in der Spenderhämatopoese nach allogener SZT und Nachbeobachtung von etwa 10 Jahren [12].

Demgegenüber besteht bei Geschwisterspender*innen aufgrund des in der Regel höheren Alters im Vergleich zu Fremdspender*innen ein höheres Risiko des Vorliegens einer CH [8]. Des Weiteren scheint eine familiäre Prädisposition ebenfalls das Risiko des Vorliegens einer Spender-CH zu erhöhen [23].

In einer großen Studie zu dieser Thematik wurden Proben von 500 Familienspender*innen (Alter ≥ 55 Jahre) zum Zeitpunkt der Stammzellspende untersucht. In 16 % aller Proben konnte eine klonale Mutation gefunden werden. Die longitudinale Untersuchung von 22 Patient*innen, die ein Transplantat mit nachgewiesener CHIP-Mutation erhalten hatten, zeigte bei fast allen Patient*innen ein Engraftment des initialen Spender-CHIP-Klons in den Empfänger*innen. Interessanterweise war bei Patient*innen mit CHIP-Transplantation eine erhöhte Rate chronischer, allerdings nicht akuter Graft-versus-Host-Erkrankungen („graft vs host disease“ [GvHD]) zu beobachten. Gleichzeitig zeigte sich eine signifikant reduzierte kumulative Inzidenz von Rezidiven bei diesen Patient*innen. Als mögliche Erklärung kann eine erhöhte Alloreaktivität durch ein CHIP-induziertes proinflammatorisches Milieu diskutiert werden, das in Einklang mit der klinisch beobachteten erhöhten Rate chronischer GvHD steht [6]. Eigene Daten zeigen, dass der Prozess des epigenetischen Alterns von Spenderblutzellen nach Transplantation teilweise beschleunigt verläuft und dass dies ebenfalls im Zusammenhang mit einer chronischen GvHD zu stehen scheint. Eventuell wird dies auch bedingt durch den differenziellen Selektionsvorteil von Klonen mit bestimmten Alterationen [25].

### Einfluss von GvHD und GvHD-Prophylaxe

Im Kontext eines möglichen Einflusses der CH auf Überleben und Rate an chronischer GvHD nach allogener Transplantation stellt sich die Frage, welche Rolle die Wahl der Immunsuppression nach allogener SZT spielen kann. Die derzeit größte publizierte Studie zu dieser Thematik hat den Einfluss von Cyclophosphamid nach allogener SZT, einem gängigen Protokoll beispielsweise im Rahmen haploidenter Transplantationen, untersucht. Hierbei konnte gezeigt werden, dass das Vorliegen einer *DNMT3A*-Mutation im Transplantat mit einem reduzierten Rezidivrisiko und einer signifikant höheren Rate chronischer GvHD assoziiert ist. Dieser Effekt zeigte sich allerdings nicht bei Verwendung von Cyclophosphamid zur GvHD-Prophylaxe. Dieser Umstand könnte in Zukunft im Hinblick auf die Wahl der Immunsuppression bei bestimmten Erkrankungscharakteristika bedeutsam sein [9].

### Resümee

Die CH im Kontext einer allogenen SZT stellt einen, wenngleich sehr seltenen, Risikofaktor für die Entwicklung einer Spenderleukämie dar. Die Wahrscheinlichkeit des Nachweises einer CH hängt vom Alter der Spender*innen und der verwendeten Sequenzierungstiefe ab. Ein generelles Screening auf CH in Spender*innen findet bisher nicht statt und hat bislang auch keinen Einfluss auf die Auswahl der Spender*innen.

## Klonale Hämatopoese im Kontext der CAR-T-Zell-Therapie

**Figure 2:**
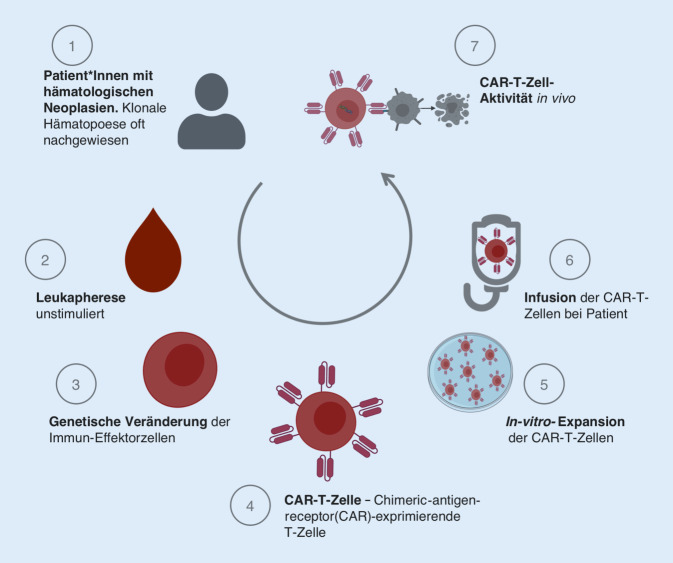

Bei den aktuell zugelassenen und in der klinischen Routine eingesetzten CAR-T-Zell-Produkten handelt es sich ausschließlich um autologe CAR-T-Zell-Präparate. Im ersten Schritt der Herstellung sind eine nichtstimulierte Leukapherese des peripheren Bluts und eine Extraktion der entsprechenden Immuneffektorzellen, in diesem Fall T‑Zellen, notwendig. Die nachfolgenden Herstellungsschritte beinhalten dann unter anderem eine Transduktion und In-vitro-Expansion der Immuneffektorzellpopulation, bis das fertige CAR-T-Zell-Produkt den Patient*innen reinfundiert werden kann (Abb. 2).

Sowohl Wirkung als auch Nebenwirkung einer CAR-T-Zell-Therapie sind eng mit einer Inflammationsreaktion verknüpft. Diese wird maßgeblich über myeloide Bystander-Zellen und die Aktivierung proinflammatorischer Zytokinkaskaden, insbesondere unter Beteiligung von Interleukin‑1 und Interleukin‑6, reguliert. Es ist bekannt, dass das Vorliegen von CH in myeloischen Zellen die Inflammationsreaktion verstärken und Interaktionen zwischen Immunzellen verändern kann [2, 15, 18]. Darüber hinaus können auch die transduzierten Immunzellen selbst durch eine CH direkt beeinflusst sein. Vor allem Mutationen in *TET2* und *DNMT3A*, die beide zu den häufigen CH-Mutation zählen, haben in diesem Kontext eine übergeordnete Bedeutung und können die CAR-T-Zell-Behandlung beeinflussen [5, 20].

Bisher existierende Daten zeigen eine durchgängig hohe Prävalenz von CH vor CAR-T-Zell-Therapie

Im klinischen Kontext ist neben einer veränderten Aktivität und Effektivität der CAR-T-Zell-Therapie vor allem ein möglicher Einfluss auf die inflammationsassoziierten Nebenwirkungen bedeutsam. Hierzu zählen maßgeblich

- das Zytokinfreisetzungssyndrom („cytokine release syndrome“ [CRS]),
- Neurotoxizität („immune effector cell-associated neurotoxicity syndrome“ [ICANS]) und
- eine prolongierte Zytopenie nach CAR-T-Zell-Therapie.

Die bisher existierenden Daten zu dieser Thematik zeigen eine durchgängig hohe Prävalenz von CH vor einer CAR-T-Zell-Therapie (34–48 %), was unter anderem auf die intensive Vorbehandlung dieser Patient*innen zurückgeführt werden kann. Darüber hinaus fand sich in einer longitudinalen Analyse von Patient*innen nach CAR-T-Zell-Therapie häufig eine klonale Progression im Langzeitverlauf, was in diesem Kontext für die Entwicklung sekundärer myeloischer Neoplasien von Bedeutung sein könnte [26]. Hinsichtlich der klinischen Nebenwirkungen zeigen erste größere Kohortenanalysen eine erhöhte Rate an schweren CRS-Verläufen bei jüngeren Patient*innen < 60 Jahren mit Nachweis von CH vor CAR-T-Zell-Behandlung [16]. Auch für die immuneffektorzellassoziierte Neurotoxizität (ICANS) ist kürzlich erstmals eine Assoziation von CH und schweren Verläufen beschrieben worden [21]. Inwiefern die CH zur Pathogenese der Zytopenie nach CAR-T-Zell-Therapie beiträgt, ist noch unzureichend untersucht [22, 26]. Im Gegensatz zur autologen Transplantation scheint das Vorliegen einer CH im Rahmen einer CAR-T-Zell-Therapie bisher nicht mit einem schlechteren Überleben assoziiert zu sein.

Die bisherigen Daten beziehen sich nahezu ausschließlich auf Untersuchungen aus dem peripheren Blut der Patient*innen vor Reinfusion des CAR-T-Zell-Produkts. Inwieweit die CAR-T-Zell-Produkte selbst und im Speziellen die CAR-transduzierten Zellen von den klonalen Veränderungen betroffen sind und welche Auswirkungen dies für das klinische Outcome der Patient*innen hat, ist zum jetzigen Zeitpunkt noch ungeklärt. Um diesen Aspekt näher beleuchten zu können, werden weitere Analysen notwendig sein, einschließlich Untersuchungen auf Einzelzellebene.

## Fazit für die Praxis

- Der Nachweis klonaler Hämatopoese (CH) ist generell abhängig vom Alter der untersuchten Personen und der Sensitivität der Sequenzierungsmethode.
- Es existieren zunehmend Daten, die zeigen, dass das Vorliegen einer CH im Kontext einer zellulären Therapie mit komplexen Interaktionen verbunden ist. Die Auswirkungen auf die einzelnen Verfahren sind hierbei divergent und bisher noch unzureichend charakterisiert und verstanden.
- Das Vorliegen von CH kann das Risiko der Entwicklung sekundärer myeloischer Neoplasien bei Patient*innen, die sich einer zellulären Therapie unterziehen, erhöhen.
- Für die klinische Praxis ergeben sich allerdings zum aktuellen Zeitpunkt keine weiteren Konsequenzen, weder für die Durchführung von Screeninguntersuchungen bei Patient*innen oder Spender*innen noch für Änderungen des Vorgehens bei Nachweis einer CH.
